# 2D Nanomaterial-Based Surface Plasmon Resonance Sensors for Biosensing Applications

**DOI:** 10.3390/mi11080779

**Published:** 2020-08-15

**Authors:** Sachin Singh, Pravin Kumar Singh, Ahmad Umar, Pooja Lohia, Hasan Albargi, L. Castañeda, D. K. Dwivedi

**Affiliations:** 1 Amorphous Semiconductor Research Lab, Department of Physics and Material Science, Madan Mohan Malaviya University of Technology, Gorakhpur 273010, India; sachin111iitp@gmail.com (S.S.); singhpraveen203@gmail.com (P.K.S.); 2 Department of Chemistry, Faculty of Science and Arts, Najran University, Najran 11001, Saudi Arabia; 3 Promising Centre for Sensors and Electronic Devices (PCSED), Najran University, Najran 11001, Saudi Arabia; albargih@yahoo.com; 4 Department of Electronics and Communication Engineering, Madan Mohan Malaviya University of Technology, Gorakhpur 273010, India; lohia.pooja6@gmail.com; 5 Department of Physics, Faculty of Science and Arts, Najran University, Najran 11001, Saudi Arabia; 6 Sección de Estudios de Posgrado e Investigación de la Escuela Superior de Medicina, Instituto Politécnico Nacional, Plan de San Luis y Díaz Mirón S/N, Casco de Santo Tomas, Alcaldía Miguel Hidalgo, C.P. 11340 Cd. de México, Mexico; lcastaneda@ipn.mx

**Keywords:** 2D nanomaterials, surface plasmon resonance sensor, dichalcogenides, biosensing

## Abstract

The absorption and binding energy of material plays an important role with a large surface area and conductivity for the development of any sensing device. The newly grown 2D nanomaterials like black phosphorus transition metal dichalcogenides (TMDCs) or graphene have excellent properties for sensing devices’ fabrication. This paper summarizes the progress in the area of the 2D nanomaterial-based surface plasmon resonance (SPR) sensor during last decade. The paper also focuses on the structure of Kretschmann configuration, the sensing principle of SPR, its characteristic parameters, application in various fields, and some important recent works related to SPR sensors have also been discussed, based on the present and future scope of this field. The present paper provides a platform for researchers to work in the field of 2D nanomaterial-based SPR sensors.

## 1. Introduction

Currently, the surface plasmon resonance (SPR) technology has drawn much attention around the whole world, due its versatility for optical device application. The concept of SPR came in the late 1970s for the characterization of thin film and observing the process at the metal interface [1,2,3,4,5,6]. Wood et al. (1902) wrote the first document on SPR principle and observed a new diffraction phenomenon of light [7]. Thurbeder et al. used attenuated total reflection (ATR) geometry for getting information about the thin film of metal on substrate [8]. For the generation of surface plasmons (SPs) a material is required, which is capable of generating the SPs after interaction with light. The metals like aluminium (Al), copper (Cu), gold (Au), silver (Ag), indium (In) and sodium (Na) are capable of generating the SPs when light is incident on metal surface. When the wave vectors of incident direct light and SPs match, the condition is known as SPR condition.

SPR sensor is an optical refractometer that can evaluate the change in refractive index (RI) of the medium at the SPR sensing surface. When the RI changes, the properties (e.g., wavelength, phase, dielectric constant, angle, etc.) of the sensing medium will be changed accordingly. This works on the concept of simple and exclusive optical phenomena. SPR sensors are mainly two types: propagation surface plasmon resonance (PSPR) sensors and localized surface plasmon resonance (LSPR) sensors [9]. PSPR is typically propagated continuously by prism coupling or grating on a thin metal film surface while LSPR can propagate along the metal/dielectric surface [10]. In the present paper, we are discussing the generation of surface plasmon waves (SPWs) at the metal/dielectric surface, such that the LSPR technique has an important role in N-layer structure. Noble metal nanoparticles display a broad UV–visible absorption band (UV-vis) which is not present in the bulk metal spectrum. This band of absorption occurs when the incident photon frequency resonates with the conductive electrons’ collective oscillation, and is known as the Localized Surface Plasmon Resonance (LSPR) [10].

LSPR sensing is an important technique focused on metal nanoparticles’ (NPs’) fast electromagnetic response to changes in RI in their immediate neighbourhood. As light over nanoparticles occurs, specific electronic modes can be excited to collectively move the conduction band of electrons [11]. Such resonance oscillations are also called localized surface plasmons, the nanoparticles disperse light strongly within a specific wavelength range. LSPR sensing experiments are also useful for converting the white light source into the visible spectra [12]. The scattered light is measured with a spectrometer and spectrum changes are then transformed into binding data [8]. LSPR nanoparticle-based sensors are less sensitive as compare to the SPR sensor [13,14,15,16,17,18,19]. The sensitivity range comparison of SPR and LSPR is shown blow in Table 1.

**Figure 1:**
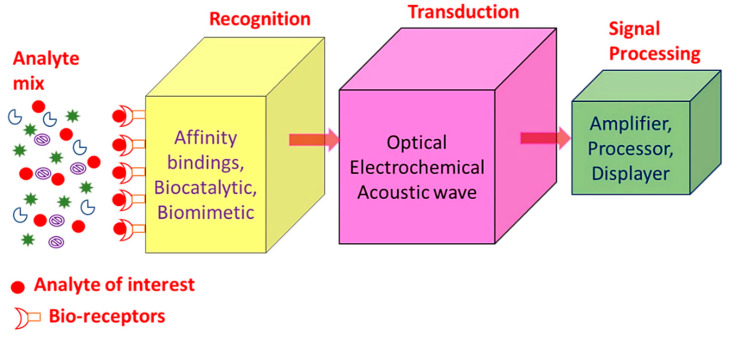
Summary of the different biosensor components.

SPR sensors, i.e., gold, copper or silver planar films have been utilized for around 30 years as refractive index-based sensors to detect the binding of analytes on or near a metal surface. This has been commonly used to detect a wide range of analyte–surface binding interactions including small molecular adsorption, ligand–receptor binding, self-assembled monolayer protein adsorption, antibody–antigen binding, the hybridization of RNA and DNA, and DNA–protein interactions. Just as in LSPR spectroscopy, SPR spectroscopy’s sensing mechanism depends on the minor changes in the refractive index occurring in response to analyte binding at or near the surface of a noble metal (Au, Ag, Cu). Chemosensors and biosensors based on SPR spectroscopy have many desirable properties as shown in Table 1 [20,21,22]. A biosensor is a device which enables the detection and analysis of biomolecules. Biosensors are used in many applications like drug production, disease prevention and pollutants’ detection, etc. Figure 1 displays a component of biosensor; it consists of the following:

- **Analyte:** A material whose chemical constituents are described and measured. Glucose, for example, is an ‘analyte’ designed to detect glucose in a biosensor.
- **Recognition:** A recognition element also known as a bio-receptor, which is a biological element (DNA probe, enzyme, antibody, etc.) susceptible to analyte recognition (antigen, complementary DNA, enzyme substrate, etc). It is important for the bio-receptor to be directly sensitive to the target analyte in order to avoid interference from certain signal sources or substances from the sample matrix.
- **Transducer:** The transducer is a component which converts one energy source into other. Inside a biosensor, the function of the transducer is to turn the bio-recognition event into an observed signal. This cycle of energy transfer is known as signalling. Some transducers emit optical or electrical signals that are typically proportional to the amount of analyte-bio-receptor interactions.
- **Signal processing:** The work of the signal-processing unit is to process the transduced signal and prepare it for display. It consists of complex electronic circuitry conducting signal conditioning, such as analogue-to-digital amplification and signal transfer. The interpreted signals are then quantified via the display device with the biosensor. The display has a user interpretation system, such as a liquid crystal display on a computer or a direct printer, which generate numbers or curves that the user can understand. Often this part has a combination of hardware and software which generates user-friendly biosensor results. Depending on the end user’s requirements, the output signal on the monitor may be numerical, graphical, tabular or picture [1,2,3,4,5].

The comparative refractive index sensitivities are significant differences to understand between the SPR and LSPR sensors. SPR sensors display great sensitivities of the refractive index (~10^6^ nm/RIU). The SPR response is frequently recorded as a shift in refractive index units, for this reason. In comparison, the LSPR nanosensor has a moderate sensitivity to the refractive index (10^2^ nm/RIU). Since this number for the LSPR nanosensor is four orders of magnitude smaller than the SPR sensor, the initial predictions estimated that the LSPR nanosensor would be 10,000 times less sensitive than the SPR sensor, and this is not the case. In reality, their sensitivities make the two sensors very competitive. The short (and tuneable) characteristic decay length of the electromagnetic field gives the LSPR nanosensor its increased sensitivity. Similar to the SPR sensor, the sensitivity of the LSPR nanosensor stems from the distance dependence of the average induced square of the electrical fields extending from the surfaces of the nanoparticles. Due to the lower refractive index sensitivity, no temperature control is required for the LSPR nanosensor, whereas the SPR sensor (with a high refractive index sensitivity) does [22]. LSPR has a simple instrumentation compared to SPR. In addition, the LSPR applications can also be adapted for portable diagnostic applications.

**Figure 2:**
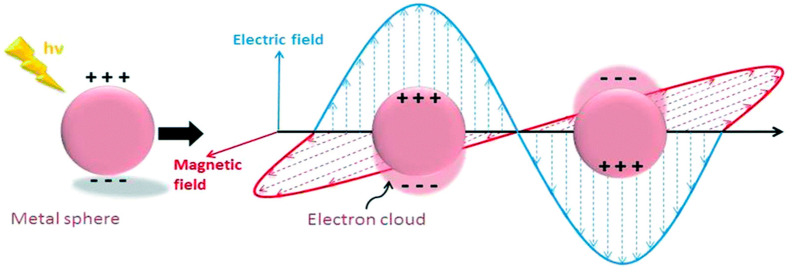
Schematic diagram of the free oscillating electrons at the nanosphere interface due to the electric field applied with arbitrary polarizations. Reprinted with reference [12].

LSPR is another mechanism for the absorption of light, which is observed within the visible light range for several metals’ NPs such as Ag, Au and Cu. It is also an optical phenomenon when a light incident on a metal NP is smaller than the wavelength of the incident light [6]. This results in a strong interaction between the electrical field incident and the free conductive electrons of the metal NPs shown below in Figure 2 [12].

The plasmon propagates across the metal–dielectric interface in propagating surface plasmon resonance (PSPR), for distances ranging from tens to hundreds of micrometres and transient decay along the z direction. The interaction between the Electromagnetic (EM) waves confined to the metal surface and the molecular interest layer causes the plasmonal resonance to shift, which can be observed in three ways: (a) imaging; (b) wavelength shift; and (c) the angle solved. The two modes, the change in wavelength and the fixed angle, are mutually related. The wavelength shift mode measures the light reflectivity from the metal surface as a function of wavelength at a constant incidence angle whereas the angle solved mode measures the wavelength change at the interface as a function of incidence angle at a constant wavelength. In imaging mode, mapping the reflectivity of the sample as a function of its position by taking the wavelength and incidence angle as constant for the light to interrogate the two-dimensional region of the sample.

The LSPR and PSPR of each technique help to find thermodynamic and real-time kinetic data for binding processes [20,21,22,23,24,25,26,27]. The PSRP technique is more sensitive than the LSPR technique when the RI shifts in bulk and both the techniques are also comparable when measuring the short-range RI changes due to the molecular adsorption layer.

**Figure 3:**
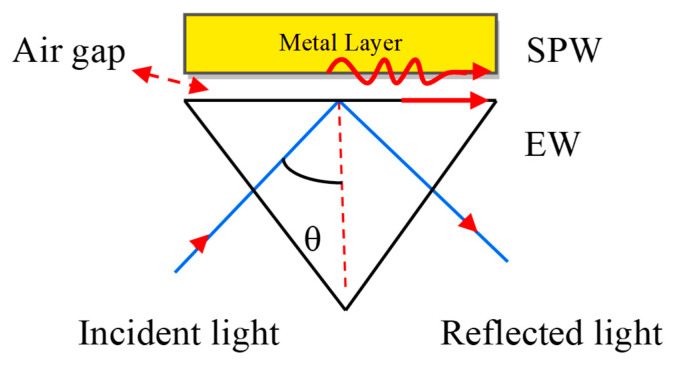
Otto configuration. Here, EW stands for Evanescent Wave and SPW refers to the Surface Plasmon Wave.

If a monochromatic light source occurs directly on the metal surface, the surface plasmon waves (SPWs) are produced on the metal–dielectric interface, but the resonance condition is not satisfied due to a lack of light accumulation on the metal surface. For the maximum accumulation of light at the metal surface, they must be coupled with a prism. The concept of coupling the metal with the prism was proposed by Otto et al. [28]. He presented a configuration shown in Figure 3. Where the metal layer is placed at the base of the prism and maintains the air gap between the metal layer and base prism.

**Figure 4:**
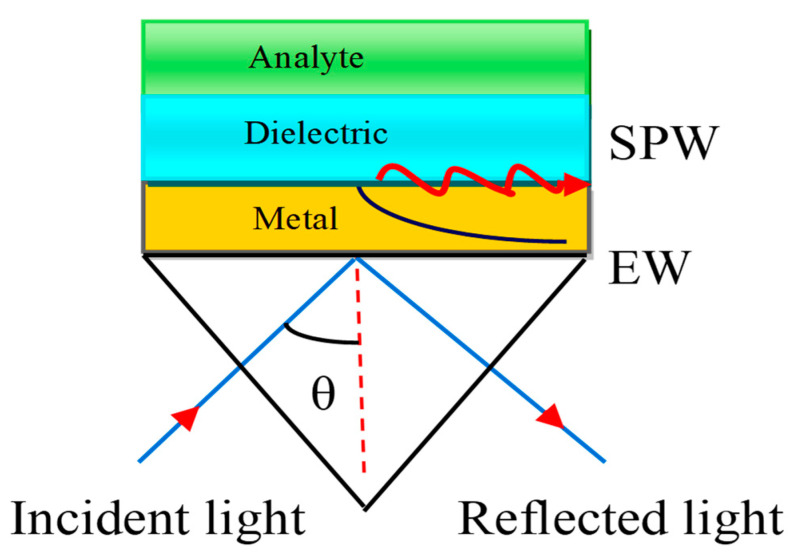
Kretschmann configuration. Here, EW stands for Evanescent Wave and SPW refers to the Surface Plasmon Wave.

The SPW is generated on the surface of the air–metal interface. The associated evanescent wave (EW) decreases exponentially from the air to metal. It is therefore difficult to sustain the air gap between the prism and metal layer. E. Kretschmann et al. [29] improved the Otto configuration as depicted in Figure 4.

In this configuration, the metal layer having a thickness of 10–100 nm is deposited on the prism base by the glue-gel whose refractive index (RI) matched both the prism and the metal refractive index.

Therefore, there is no air gap between the metal layer and the prism. The Kretschmann configuration is a very useful technique to improve the sensitivity of the SPR sensor. The associated EW decreases exponentially in both media, such as the metal and dielectric media. The dielectric layer is also connected with a sensing medium or analyte in which the biomolecules are to be detected.

The generation of surface plasmons depend upon the method of optical excitation. There are various optical excitation methods present, such as the prism, grating and wavelength coupling method etc. The present paper focused on a prism coupling method, working on the principle of the ATR technique [14]. The prism coupling technique is a very famous technique utilized in Kretschmann configuration. The first demonstration of SPR for gas sensing was carried out in 1983 by Liedberg [5], after which the SPR became the most approved sensing technology.

Based on the Kretschmann configuration, some other nanomaterials are also being used to enhance the sensitivity of the SPR sensor, such as two-dimensional (2D) nanomaterials. In the last two decades, 2D nanomaterials such as graphene, black phosphorous (BP) and transition metal dichalcogenides (TMDCs), i.e., MoS_2_, MoSe_2_, WS_2_, and WSe_2_, have gained great attention for the sensitivity enhancement of SPR biosensors. It has become a current topic of research due to the exclusive optical and electrical properties used in biosensing applications [30]. Since 2D nanomaterials have unique properties, a robust development framework for various system components can be developed for different device components such as batteries, transistors, detectors, ultrafast lasers, optoelectronic components, biological and physical sensors, photovoltaic cells, etc. There have been thousands of research articles published on 2D nanomaterials (graphene, BP and TMDCs) in different fields in the last decade.

Among these 2D materials, the most commonly used material is graphene because it hardly and stably adsorbs biomolecules due to the pi (π) stacking interaction between hexagon cells, similarly in biomolecules, where the carbon-based ring structures are extensively present [31]. Graphene shows various properties such as the high surface to volume ratio, fast electron mobility, the balanced structure between graphene layers, indirect bandgap, highly optically transparent (97.7%), etc. The property’s high surface area to volume ratio is very useful to enhance the sensitivity of the SPR sensor. Wu and Chu et al. proposed a graphene-based highly sensitive SPR sensor which achieved a 25% enhancement of sensitivity [31].

TMDCs semiconductor group materials are known as non-graphene 2D nanomaterials. Compared to the zero-bandgap energy of graphene, they provide an invisible spectrum of dichalcogenide transition metal near IR, such as MX_2_, where (M = X = S, Se; Mo, W). Such materials have been shown to have clear bandgaps in the monolayer limit, a property well suited for applications in photonics and optoelectronics. 2D semiconductor TMDCs’ have tremendous electronic and optical properties, focusing on heavy excitonic effects and properties depending on spin and valley.

TMDCs consisting of a single layer of transitional metal atoms in a trigonal prism, are sandwiched between two layers of chalcogen atoms. Bulk TMDCs are indirect-bandgap semiconductors with a minimum conduction band and maximum valence band position, respectively [32,33]. They were shown to cross over in the monolayer limit to become the direct-bandgap semiconductors with gaps at the points K and K’. This trend can be interpreted as a consequence of an expanded indirect gap due to the substantial impact of out-of-plan quantity containment, although the direct gap at points K and K’ remains relatively uninfluenced [34,35]. Table 2 summarizes the basic electronic characteristics of the various TMDCs, measured both experimentally and theoretically.

**Figure 5:**
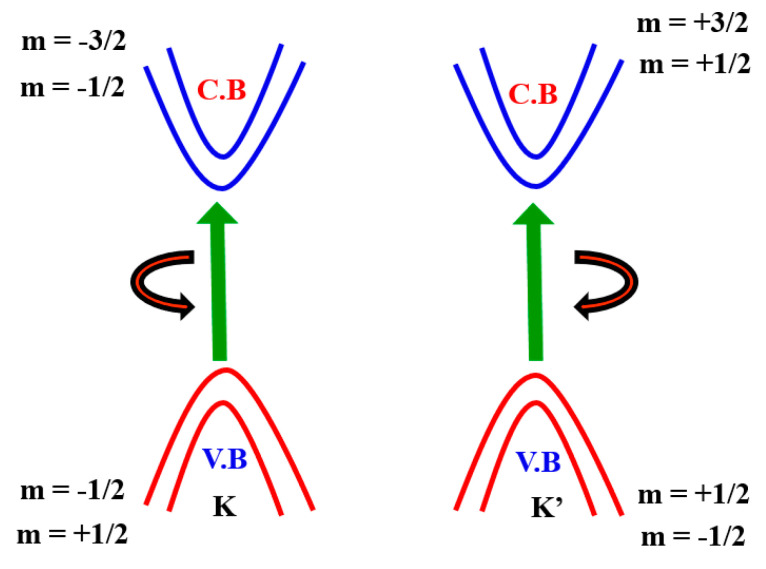
Electronic band structure of spin–orbit interaction around the point K and K’.

The monolayer TMDCs’ electronic structure near Fermi energy defines two important properties of materials—electrical and optical properties—which can be defined by two copies of degenerate valence bands and conduction band around the K and K’ points (Figure 5). This is similar to that of a gaped graphene with a broken inversion symmetry and can be modelled with the degree of freedom of spin and valley pseudospin (K or K’) by an efficient 2D massive Dirac Hamiltonian. The spin-up condition in the K Valley is degenerated by the spin-down condition in the K’ valley. (Figure 5). As several groups have shown experimentally that degeneration can be removed by applying an out-of-plane magnetic field to break the time-reversal symmetry [61,62,63,64], TMDCs in the K and K’ valleys yield nearly finite Berry curvature and orbital magnetic moments (m), i.e., the broken TMDC inversion symmetry. Specifically, the magnetic moment allows for the combination of a pseudospin valley with the magnetic field as stated above and gives rise to a valley-dependent optical theoretical framework [65,66,67,68]. Here, the azimuthal total quantum orbital is represented by m and it is also known as an angular spin momentum. Hence, the electrical transport properties are also valley-based.

In the near-infrared and visible spectral regions, the optical absorption of monolayer TMDCs is dominated by direct transitions between valence and conduction bands around points K and K’ [66,67,68,69]. Direct band-to-band transitions in 2D are usually characterized by a step-function-like continuum from the energy-independent joint-density-of-state and transition matrix elements near to the edges of the parabolic band in the absence of excitonic effects. The large binding energies have been verified by recent experiments based on optical spectroscopy [68,69,70] and tunnelling spectroscopy scanning [71,72,73], although there are discrepancies in the detailed EB values from various experiments and interpretations (Table 2).

The extremely powerful excitonic effects have significant influence on the optical properties of 2D TMDCs. For the photonic application of 2D TMDCs, the broad exciton binding strength, high absorbance and short radiative lifetime play a vital role. Such materials have a clear bandgap, which means that the applications are not limited to nanotechnology and opto-electronics but have a wide potential for sensor technology. Perhaps the most auspicious nominee for optical and electronic applications is the TMDC-based nanomaterial MoS_2_. MoS_2_ has a high rate of absorption (5.6%) compared with graphene sheet monolayer (2.3%) [74,75,76,77,78,79,80]. The 2D material-based SPR sensor heterostructure is also used to improve the sensitivity. The present paper deals with the development and current status of Kretschmann configuration-based SPR sensors using 2D material, including SPR sensing principle, matrix-based theoretical and mathematical modelling, characteristic parameters, and material selection importance with some applications.

## 2. Fundamentals of SPR Sensing

**Figure 6:**
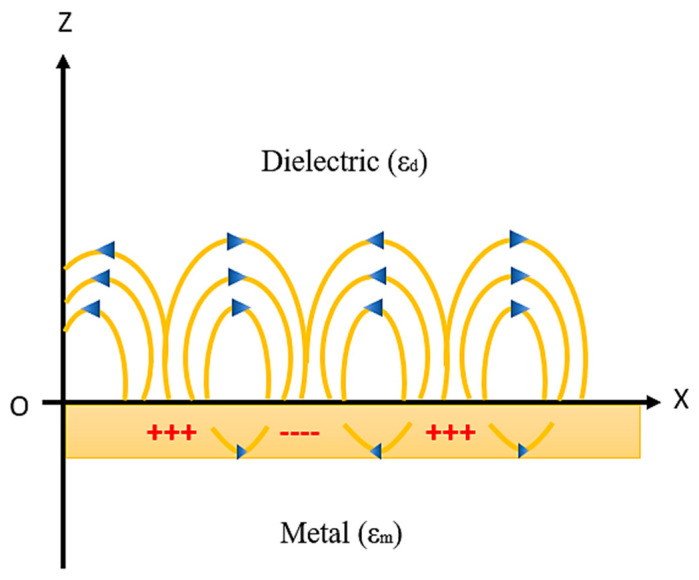
SPW generation at the metal–dielectric interface. Idea adopted from reference [82].

The principle of generation of SPR on the metal–dielectric interface and the resonance condition will be explained in this section. Whenever a metal and dielectric medium comes together on the same interface then the charge density oscillation exists, which is called surface plasma oscillations [78,79,80,81,82,83,84,85] and the corresponding propagated waves are known as surface plasmon waves (SPW’s). This phenomenon takes place when a monochromatic light source passes through the prism to the metal–dielectric interface, thus surface plasmon waves are generated as shown in Figure 6.

SPWs are longitudinal waves (i.e., p-polarized) generated on the metal–dielectric interface when light passes through the metal. Both the metal and dielectric sample consist of complex RI nm = $\sqrt[{2}]{\in_{m}}$ and n_d_ = $\sqrt[{2}]{\in_{d}}$, respectively, as shown in Figure 6. The surface plasmon propagates in parallel to the x direction as an electromagnetic wave with a magnetic field directed in parallel to the y direction, that is, the transvers magnetic polarization (TM) state. 

For the TM polarization state, it is required to generate charge distribution on the metal surface, which is the first required condition for SP excitation. The boundary value problem condition for Maxwell’s equation helps to explain the phenomenon of surface plasmon. Now, considering the TM plane and electromagnetic fields generated at the interface with appropriate waves:

(1) $$E_{i\;\left(r,t\right)=\;\left({\;E}_{\mathrm{ix}\;},0\;,{\;E}_{\mathrm{iz}}\right)}[e^{-\left|Z\right|k_{\mathrm{iz}\;}}{\;e}^{i\left({\mathrm{xk}}_{\mathrm{ix}}-\mathrm{wt}\right)}]$$

(2) $$H_{i\;\left(r,t\right)=\;\left(\;0\;,{\;H}_{\mathrm{iy}}\;,\;0\;\right)}[e^{-\left|Z\right|k_{\mathrm{iz}\;}}{\;e}^{i\left({\mathrm{xk}}_{\mathrm{ix}}-\mathrm{wt}\right)}]$$

where, (i = metal, dielectric), k_zi_ and k_ix_ are the z and x components of the wave vectors. Substituting the field from Equations (1) and (2) and applying continuity conditions then we get resultant relation:

(3) $$K_{zi}=\;\sqrt{\varepsilon_{\;i\;}{\left(\frac{\omega }{c}\right)}^{2}-k_{\mathrm{ix}}^{2}}$$

and

(4) $$\frac{k_{z1}}{\varepsilon_{m}}{\;H}_{1y\;+}\frac{k_{z2}}{\varepsilon_{d}}{\;H}_{2y}\;=\;0$$

H_1y_ − H_2y_ = 0 (5)

Solving the above equations by equating the determinant equal to zero leading to:

(6) $$k_{z1}\varepsilon_{d\;+}k_{z2}\varepsilon_{m\;}=\;0$$

Under the phase matching condition K_1x_ = K_2x_ = K_x,_ the resultant expression is found:

(7) $$K_{x}\;=\;\frac{\omega }{c}\sqrt[{2}]{\frac{\in_{m}\in_{d}}{\in_{m}+\in_{d}}}$$

Equation (7) represents the surface plasmon wave vector K_x_ = K_sp_ and defines the dispersion relation for the generation of surface plasmon.

Where $\in_{m}$ and $\in_{d}$ are the dielectric constants of the metal and dielectric layer with the frequency (ω) and velocity of light (c), metal contains a complex dielectric constant value; the real part provides the effective RI while the imaginary part indicates the attenuation in the direction of propagation of SPW.

Since the light propagate through the dielectric medium, the relation between the propagation constant ($K_{d}$) and dielectric constant ($\in_{d}$) is given by

(8) $${\;K}_{d}=\frac{\omega }{C}\sqrt[{2}]{\in_{d}}$$

Equations (7) and (8) suggest that K_sp_ ˃ ${\;K}_{d}$ because $\in_{m}$ ˂ 0 and $\in_{d}$ ˃ 0 at a given frequency i.e., the direct light cannot excite the surface plasmon at the interface, for excitation extra energy and momentum must be required for a light wave in a dielectric medium. Therefore, different coupling methods can be used for the optical excitation of surface plasmon like prism coupling, grating coupling, wave guide coupling methods, etc. In the present paper, we use a prism coupling method to excite the surface plasmon from the interface for satisfying the SPR condition, and it is satisfied if the surface plasmon wave vector can be excited in dielectric medium. The surface plasmons are excited if EW propagate along the interface in place of direct light. A prism of high refractive index generates EW [81,82,83].

**Figure 7:**
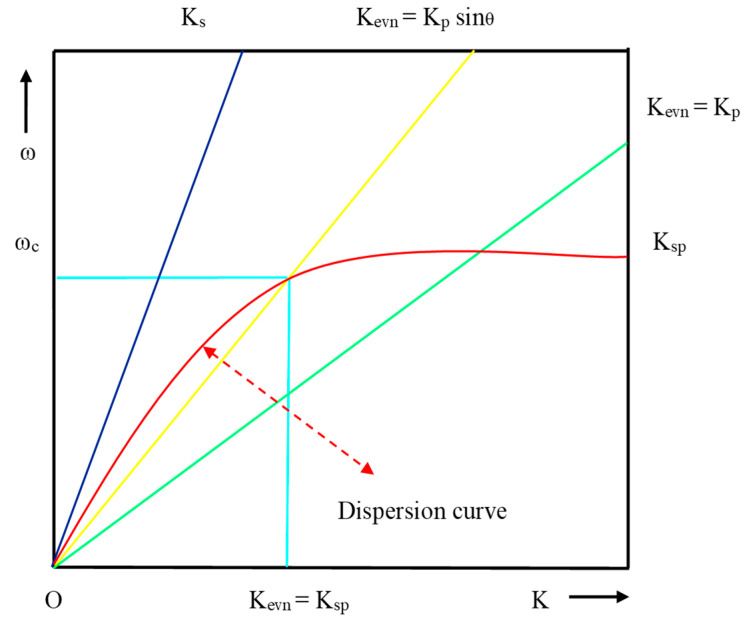
Dispersion curve for direct light, EW and SPW.

E. Kretschmann and H. Reather [29] designed a prism-based structure, shown in Figure 7, to excite the surface plasmon using EM. In this structure, a thin film of metal layer coated on the base of the glass prism and back side of metal layer kept in touch with the dielectric medium of lower RI. When p-polarized light occurs at an angle (θ) greater than the critical angle required for total internal reflection (TIR) on the prism–metal layer, an EW is generated at the interface of the prism–metal layer but it also exponentially decays. The propagation constant (K_evn_) of EW related with the dielectric constant ($\in_{p}$) of prism is given by

(9) $$K_{\mathrm{evn}}\;=\;\frac{\omega }{C}\sqrt[{2}]{\in_{p}}\sin\theta \;=\;K_{p}\sin\theta$$

Form resonance condition, K_sp_= K_evn_ and θ = θ_res:_

(10) $$\frac{\omega }{\;C}\sqrt[{2}]{\in_{p}}\;\mathrm{Sin}\theta_{\mathrm{res}}\;=\;\frac{\omega }{C}\sqrt[{2}]{\frac{\in_{m}\in_{d}}{\in_{m}+\in_{d}}}$$

where θ_res_ is the resonance angle, defined as the angle at which the resonance condition must be satisfied for a prism of fixed RI and the dispersion curve of EW crosses the dispersion curve of SPW as shown in Figure 7.

From the dispersion curve, the resonance condition of SPR can be explained for the surface plasmon, direct light and light through the prism. EW and SPW crosses each other at the number of points for the different set of incident angle, which is lies between K_evn_ = K_p_ Sinθ and K_evn_ = K_p_. From Figure 7, it is evident that the propagation constant of SPW at the metal–dielectric interface entirely matches with the propagation constant (K_p_) of EW (K_evn_) at a certain frequency and angle of incident, and the corresponding condition is known as the surface plasmon condition.

## 3. Theoretical and Mathematical Modelling

### 3.1. Theoretical Modelling

**Figure 8:**
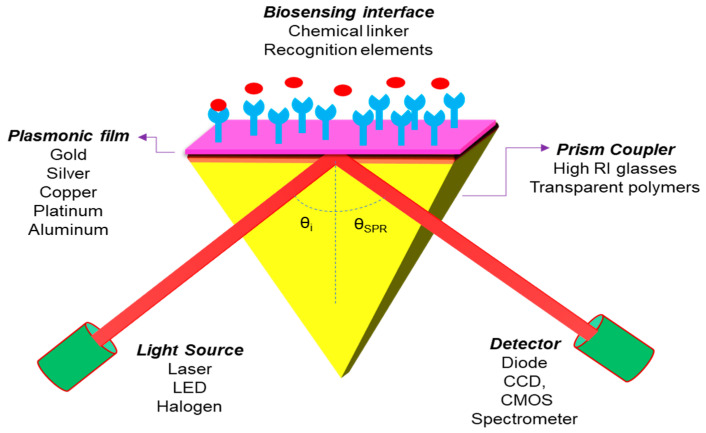
Sketch diagram for the Kretschmann configuration of an SPR sensor.

The proposed diagram of the N-layer Kretschmann Configuration for the 2D material layer-based SPR sensor is shown in Figure 8. An incident light is guided through a prism onto an SPR sensor chip and the reflected beam is detected through a photodetector or imager. The incident light excites the surface plasmons in the sensor chip (metal film) at an appropriate angle (resonance angle), and the strength of the reflected light drops to a minimum. SPR’s electromagnetic field penetrates the fluidic medium, and molecular binding processes take place on the surface, as well as changes in the refractive index in the fluidic medium.

This sensor structure covered four layers of different materials, in which Gold (Au) or Silver (Ag) film is installed as a metal layer on the top of the coupling prism and the 2D material layer coated on the metal film surface for a biomolecular recognition element. Theoretically, it is observed that the thicknesses of the layers are in the nanometer (nm) range and the operating wavelength is chosen as 633 nm to get the best sensitivity result of the SPR sensor. In the present paper, we have chosen a prism fabricated by BK7 glass, whose refractive index (RI) can be measured by using the following equation [16]. Photo-thermo-refractive (PTR) glasses are also a potential candidate for the development of high performance SPR sensors for biomedical and industrial applications as well [84,85,86]:

(11) $$n^{2}\;=\;[1\;+\;\frac{1.03961212{\;\lambda }^{2}}{\lambda^{2}-0.006000698{\;\lambda }^{2\;}}+\;\frac{0.231792344{\;\lambda }^{2}}{\lambda^{2}-0.020017914{\;\lambda }^{2\;}}\;+\frac{1.01046945{\;\lambda }^{2}}{\lambda^{2}-0.0103560653{\;\lambda }^{2\;}}\;]$$

The value of RI of the 2D material (graphene) can be determined as the given equation:

(12) $$ng_{\;}=\;3\;+\;i\;(\frac{C}{3})\;\lambda$$

where the value of constant C is 5.45 µm^−1^.

The Drude–Lorentz Model is useful to determine the optical response and RI of the Ag or Au [66]:

n^2^_m_ = ε_m_= [1 − λ^2^λ_c_/λ_p_^2^(λ_c_ + i λ)] (13)

where λ, λ_c_ and λ_p_ represent the operating wavelength of the monochromatic source of light, collision wavelength and plasma wavelength, respectively. The values of λ_c_ and λ_p_ for Ag are 176.14 nm and 145.14 nm, while for Au, they are 8934 and 168.26 nm, respectively.

The RI of each material’s wavelength (λ) 633 nm is taken at the operating point because the optical nonlinearity can be improved at a higher frequency and overall sensitivity of the sensor can be achieved at low frequency with minimum Kerr effect [87,88,89,90]. Hence, the chosen operating wavelength is 633 nm. The RIs of various glass prisms at the operating wavelength of 633 nm are shown below in Table 3.

The investigation of the experimental modulation is used to obtain the RI for the 2D material, composed of real and imaginary parts. The expression n(k) = 1.33 + Δn_a_ represents the complex refractive index of the sensitive medium and Δn_a_ represents the change in refractive index of sensing medium as a result of the biological or chemical reaction.

### 3.2. Mathematical Modeling

The transfer matrix method was used to study the reflectivity of the N-layer Kretschmann configuration model of incident light, which is precise and without approximations. The MATLAB (Natick, MA, USA) software is used to measure the analogous SPR modulation. From the N-layer model, all the layers are applied in the vertical direction (*Z* axis) of BK7 glass-coupling prism.

The refractive index (RI) and permittivity of the Nth layer are represented by n_k_ and ε_k,_ respectively. The angle of resonance has a minimum reflectance corresponding to the angle of the incident. At the first edge, the Z = Z_1_ = 0 to the last edge Z = Z_N−1_ the tangential components of the fields are continuous [90]:

(14) $$\left(\begin{array}{c} U_{1}\\ V_{1} \end{array}\right)\;=\;M\;\left(\begin{array}{c} U_{N-1}\\ V_{N-1} \end{array}\right)$$

where (U_1_, V_1_) and (U_N−1_, V_N−1_) represent the borderline terms of the first layer and N^th^ layer of electric and magnetic fields at the limiting surface. The composite architecture of the characteristic matrix is denoted by M_k_ and for parallel (p), polarized light can be written by the following Equations (14)–(20) [91]:

(15) $$\overset{N-1}{\underset{K=2}{\prod }}M_{K}=\;\left(\begin{array}{cc} M_{11} & M_{12}\\ M_{21} & M_{22} \end{array}\right)$$

where:

(16) M K = (cosβk−isinβkqk−iqksinβkcosβk)

(17) $$q_{k}\;=\;\left(\sqrt[{2}]{\frac{\mu_{k}}{\delta_{k}}}\right)\cos\theta_{k}\;=\;\frac{\sqrt[{2}]{\in_{k-n^{2}{}_{1\;}\sin\theta_{1}^{2}}}}{\in_{k}}$$

(18) $$\beta_{k}=\frac{2{\pi n}_{k}}{\lambda }\cos\theta_{k}\;(Z_{k}\;-\;Zk_{-1})\;=\;\frac{2{\pi d}_{k}}{\lambda }\;(\sqrt[{2}]{\in_{k-n^{2}{}_{1\;}\sin\theta_{1}^{2}}}\;)$$

With the help of the above equations, we found the complex reflection coefficient ($r_{p})$ of the N-layer P-polarized light:

r_p_ = [(M_11_ + M_12_q_5_) q_1_ − (M_21_+ M_22_q_5_)/(M_11_ + M_12_q_5_) q_1_ + (M_21_+ M_22_q_5_)] (19)

The amplitude reflection coefficient ($R_{p}$) could also be obtained by the expression:

(20) $$R_{p}\;=\;\left|{\sqrt[{2}]{r}}_{p}\right|$$

## 4. Characteristic Parameters

The characteristics of an SPR sensor depend on three main parameters: the sensitivity of the sensor (S), detection accuracy (DA), and the quality factor (QF); however, another important factor is full width half maximum (FWHM). All characteristic parameters should have high possible values, but a smaller FWHM, to achieve a good performance from any SPR sensor.

The parameter sensitivity is defined as the ratio of resonance angle change ($\Delta \theta_{\mathrm{res}}$) with RI variation ($\Delta n_{a}$) in analyte. The dimension of sensitivity is measured in (°RIU^−1^) [92] and calculated through Equation (15):

(21) $$S\;=\;\frac{\Delta \theta_{\mathrm{res}}}{\Delta n_{a}}$$

The dictation accuracy is defined as the resonance angle change ($\Delta \theta_{\mathrm{res}}$) ratio with FWHM. It is a dimensionless quantity and estimated by Equation (16). The dictation accuracy is also called signal to noise ratio (SNR) [93]:

(22) $$\mathrm{DA}\;=\;\frac{\Delta \theta_{\mathrm{res}}}{\mathrm{FWHM}}$$

The third important parameter is the quality factor that can be determined by the sensitivity ratio (S) to FWHM and is estimated by Equation (17) [94]:

(23) $$\mathrm{QF}=\frac{S}{\mathrm{FWHM}}$$

Sensitivity is an important parameter for an SPR-based sensor, which is also utilized in angular interrogation method. The sensitivity depends on the amount of change in the resonance angle with a change in RI of the sensing medium. The reflectance plot (R) is a function angle of the light incident beam for the two different sensing refractive indices n_m_ and n_m_ + δn_m_, and it is observed that the RI of the sensing medium increases by δn_m_ when the resonance angle shifts by δθ_res_. The width of the SPR spectrum is also responsible for detection accuracy. A narrower width provides a higher detection accuracy. The parameter which combines the two different sensitivity (or refractive indices of two different media) with detection accuracy is known as the signal-to-noise ratio (SNR).

## 5. Role of Material Selection

### 5.1. Metal Layer

Researchers are working all over the world in the field of SPR-based sensors to improve the performance. Various materials have been investigated for several decades for improving the performance. The sensitivity, quality factor, detection accuracy and operating range of a sensor are the main parameters to compare with any other SPR sensors. The best sensor has all the high value of parameters for giving reproducible results. For a SPR sensor, the performance parameters depend on the material selection. In the present paper, SPR sensor has a metal layer and 2D material layer. The metal consist complex dielectric constant (ε_n_ + iε_k_). The real part (ε_n_) and imaginary part (ε_k_) of the dielectric constant explain the reflection and absorption, respectively. The SPR spectrum also depends on the real and imaginary parts of the dielectric constant. The sharpness of the dip increases if the ratio (ε_n_/ε_k_) increases. The dielectric constant and the ratio of real and imaginary parts of different metals are listed in Table 4 [95,96,97,98,99].

From Table 4, Ag has the highest value of the ratio (ε_n_/ε_k_) among Au, Cu and Al. This means that the sharpest dip is produced by Ag, but the Ag and Cu layer films are not stable due to oxidation phenomena. Thus, the performance of the SPR sensor is reduced and it is difficult to give a reliable result for practical application. Au and Al are being used as metal layers in an SPR sensor, the value of ratio (ε_n_/ε_k_) for Au is higher than Al but the cost is too high for practical application. Sodium (Na) and indium (In) metal are also being used as a metal layer, but sodium is more reactive in nature and indium is very costly [100,101]. Further additional dielectric layers such as TiO_2_, SiO_2_, Ta_2_O_5_, and MgF_2_ may also be used for enhancing the performance of SPR-based sensors and waveguide-based SPR devices [102,103,104].

### 5.2. 2D Material Layer

2D materials such as graphene (G) and TMDCs, i.e., MoS_2_, MoSe_2_, WS_2_, and WSe_2_, have been the current topic of research for SPR sensors due to their unique optical and electrical properties [104], and sensors involving such materials have been used in a number of applications [105,106,107].

2D materials have excellent thermal conductivity, mechanical conductivity, optically transparent, high surface area-to-volume ratio, and high elasticity. The property’s high surface area to volume ratio is very helpful to enhance the sensitivity, quality factor and detection accuracy of the SPR sensors. Zeng et al. proposed a grapheme-MoS_2_ nanostructure-based SPR biosensor with enhanced sensitivity [108]. Wu L et al. has also reported an Al-graphene-MoS2-based SPR sensor with a sensitivity of 215 °RIU^−1^ but the quality factor of SPR was only 73 °RIU^−1^ [109]. Black phosphorous (BP) has also been used to improve the SPR sensitivity. Wu et al. have proposed a TMDC–graphene–BP heterostructure-based biochemical sensor; they could also achieve with a sensitivity of 279.0 °RIU^−1^ with a bilayer of WS2 and BP [110,111,112,113]. These reports support that BP, TMDCs and graphene are good materials for enhancing all the parameters of SPR sensors.

From the above Table 5, 2D materials have large real part of dielectric constant, thus they have a stronger ability for the absorption of the light energy [114,115,116]. The coating of 2D materials on the metal surface helps protect the metal from oxidation as a protective layer [117,118]. These advantages assist to develop the 2D material-based SPR sensor. The sensitivity of the different 2D material-based SPR sensors is compared in Table 6.

Table 7 shows the effective parameters which affect the final product as well as the advantages and disadvantages of each method [96,97].

### 5.3. Selection of Glass Prism

In SPR sensors, the selection of material for the prism is usually assessed with respect to resonance angle tuning, with a better RI of prisms generating less of an angle of resonance. In addition, there is an additional advantage for photography with the use of large RI prisms as less incidence angles can result in small distortions in image and expanding reflecting properties over the surface. A few extensively utilized optical glasses are SF2 (n = 1.65), SF10 (n = 1.72) and BK7 (n = 1.51); each accomplishes the corresponding conditions of SPR resonance and provides an accessible incident angle for 50 nm gold films excited by visible light. Sapphire and Quartz prisms were utilized for UV–SPR [133,134] and for NIR–SPR a fluoride glass prism was tested with high sensitivity [135,136,137,138], while performing experiments in other wavelength regions. However, besides altering the incident wavelength, changing the RI of the prism generally results in the corresponding change in sensitivity, with lower RI prisms producing larger responses of the sensor. As such, the substrates of low RI prism are still under development which have the ability to increase keff (i.e., incident light effective wave vector) to match ksp (i.e., SP wave vector), therefore generating higher sensitivities. Recent attempts towards this aim used polymer prisms and low RI glass, both showing improved sensitivity to SPR over higher RI glass prisms. Though the larger SPR community also has to catch up with these resources. In one design, a silver SPR chip was mounted inside a liquid reservoir that functioned as both the measuring cell and the light coupler, with the metallic face of the chip sealed inside an inert gas chamber to prevent oxidation and interference with the samples. In comparison to conventional SPR sensors, changes in the refractive index of the sample resulted in decreases in the resonance angle that reacted as linearly negative. Considering the full width of the reflectivity curve at half-maximum, this sensor demonstrated a higher figure of merit than conventional glass prism sensors. Although it is noteworthy that this configuration involves a reduced number of optical components, one downside is that there is a need for adequate sample volume to cover the sensor chip and hold it submerged. Therefore, Lan et al. also developed an aqueous prism model in which the metallic SPR film was placed within a standard flow cell, and the liquid prism solution remained constant. This design also displayed high merit figures and brought the benefits of reduced sample volume and the ability to easily adjust the liquid prism refractive index by changing the liquid prism reservoir. This concept also showed high merit figures, and brought the benefits of reduced sample volume, as well as the ability to easily change the refractive liquid prism index by adjusting the reservoir of liquid prisms. Through the advent of new manufacturing methods, such as three-dimensional printing, a larger range of materials (e.g., glass, plastics) and revolutionary technologies would eventually lead the world to rethink how optical and instrumental design is performed [139,140,141,142].

### 5.4. Detection of SPR Sensor

Generally, there are four methods to detect SPR signals, which are intensity modulation, incident angle interrogation, incident wavelength interrogation and phase interrogation. The primary objective of intensity modulation is to monitor the reflectivity intensity shift, in particular the wavelength or the angle of incident. With regard to the array detector, this technique is useful for obtaining an image using an SPR sensor, generally known as SPR imaging (SPRi). In SPR imaging, the contrast image can be found either in photography or video in the sensing area. In incident angle interrogation, a monochromatic light source is incident to couple surface plasmons; therefore, an SPR condition from different refractive index medium is the contribution of different angles of incident. On the basis of light source type to couple the SPR, the wavelength interrogation and incident angle of SPR can be chosen. For the monochromatic light source (laser), the angular interrogation technique is needed, whereas for the polychromatic light source for the fixation of an incident angle, the wavelength interrogation technique is required. The phase interrogation method is explicitly used for a coherent monochromatic light source in SPR instruments. However, this method requires phase shift equipment, such as a lock-in amplifier. In addition, the optical configuration of this method is more complicated than the other three methodologies. This major downside is that only limited works have reported this method for SPR sensor devices, in particular for commercial products on the market [141].

## 6. SPR Application

### 6.1. SPR Biosensors

Liedberg in 1983 developed the first SPR sensor for biosensing application [10]. Because of unique properties such as high sensitivity, high accuracy, real-time monitoring and level free detection, the SPR technique has become the most popular sensing method. The advantages and disadvantages of various biosensors is shown in Table 8. Optical sensors are best known for their high sensitivity and ability to track remotely, but they can be expensive to mount and are sensitive to external physical harm. As far as the electrochemical sensor is concerned, good resolution, excellent repeatability and precision make it a good detection approach. However, the temperature changes are easily affected and the product is limited by a short shelf life before being replaced. The biosensors of acoustic wave, i.e., quartz crystal microbalance (QCM), use a mass-based transducer that detects very small changes in mass and is very useful for analytes that do not have electrically conductive properties or inflorescence signals (e.g., viruses). Thinner quartz sheets may increase the QCM frequency for increased sensitivity, but the device will be mechanically unstable and fragile [143,144,145].

In 1994, the first survey of the method of real-time bio-specific interaction analysis was demonstrated, which have been repeatedly utilized and regularly enhanced for the analysis of thermodynamic and kinetic constants of biomolecular interactions.

Biosensing explains the fundamentals of detecting the concentration of biological objects, such as viruses, bacteria, DNA, and proteins, in the scales from nanometres (nm) to micrometres (µm). This is also applicable for sensing biomolecules, biological structures and microorganisms, etc. [50]. The SPR biosensor works based on the measurement of shift in the position of reflectance dip due to binding of biomolecules on the sensor layer. Liederberg and Nylander explained the application of SPR sensor for gas detection and biosensing [10]. It is also suitable for affinity-based biosensor. Hamola et al. [118] discussed the fundamentals of SPR affinity biosensor and its current developments for future applications. The SPR biosensor also works on detection capabilities, such as specific detection, labelled detection, real-time and label-free detection. The application of the fiber-optic SPR biosensors shown in Table 9.

### 6.2. SPR Sensor for Food Quality and Safety

Bacteria detection, pathogen detection, formalin detection, presences of germs and environmental pollution demands a quick and appropriate measurement to give information about the food safety and quality for food industry and control authorities. In recent years, biosensors have been exclusively based on the SPR technology, which have been enforced for this purpose [155,156,157,158,159,160,161]. Oh et al. developed an SPR-based sensor for Salmonella paratyphoid disease, which was a bacterial infection dispersed in humans by food poisoning, causing high fever, diarrhoea, constipation and intestinal body pain [162,163,164,165].

Md. Moznuzzaman et al. has also developed a model of highly improved 2D material (graphene and MoS2)-based SPR sensor for formalin detection [166]. The fast consumption of formalin causes various health diseases like chronic cancer, headache and skin rashes, therefore the detection of formalin in food is essential, otherwise it becomes a major issue in developed countries. The proposed sensor detects the presence of formalin by using attenuated total reflection (ATR). Thus, 2D material-based SPR sensor has an excellent feature for food safety and quality sensor development.

### 6.3. SPR Sensor for Material Characterization

The main application of SPR is biosensing and bio-molecular analysis, but it is also effective to check nanostructure film deposition, such as layer-by-layer (LbL) and Langmuir–Blodgell (LB) films. The nanostructure film thickness (d) of LB and LbL can conveniently be calculated, if RI (n) is known or evaluated. When RI is unknown, the film thickness can still be calculated using two different methods: calculating SPR spectra in two different media (air and water) or using the different wavelengths of light sources, which grants to evaluate a unique value of thickness and RI [167,168,169,170,171,172]. The Wins pall software helps to determine the relation between RI and thickness for different values.

### 6.4. SPR Sensor for the Study of the Physical Quantities

SPR sensors are also applicable for the displacement and angular position measurement which can be described on the basis of SPR sensitivity to the momentum of incident light wave. Physical process happenings in different optical transducer materials were also observed for the development of SPR sensing devices, including a humidity sensor, using moisture-induced refractive index changes in absorptive thin layers and a thermo-optic effect sensor in hydrogenated amorphous silicon [173,174,175,176,177,178].

### 6.5. SPR as Chemical Sensor

**Figure 9:**
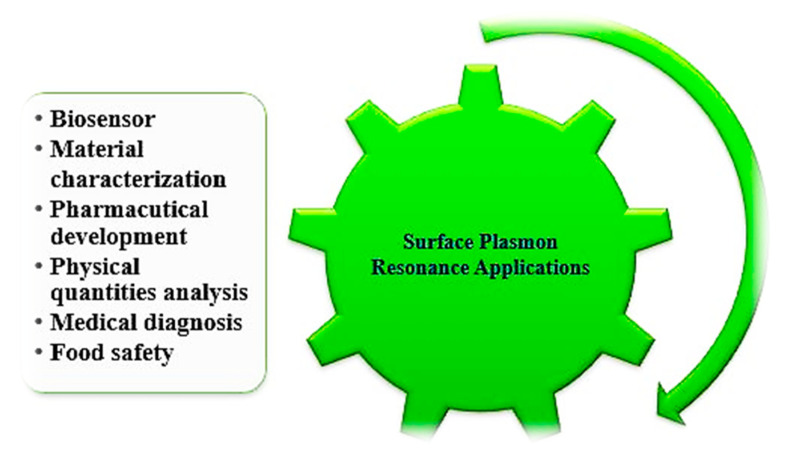
Applications of SPR sensors.

The changes in the complexity of the analyte concentration may be directly obtained by calculating the RI using an SPR sensor (e.g., monitoring distillation processes) [179,180,181,182,183,184,185], and most of the chemical SPR sensor, based on variance measurements due to adsorption, and an analyte chemical reaction with a transducer medium, result in improvements to its optical characteristics. The applications have mainly focused on change in the RI of the transducing layer induced by the adsorption of analyte molecules. The SPR sensor has additional applications in several areas, such as drug diagnostic, medical diagnostic, environmental protection, germs detection and material science, which are shown in Figure 9.

## 7. Future Perspective of SPR Sensors

Analysis and detection of chemical and biochemical substances are required in many areas like drugs and food monitoring, environmental monitoring, medicine and biotechnology. To cater to the above needs, SPR sensor technology has great potential. Currently, the SPR biosensor device is in competition with other type of biosensors [186,187,188,189,190,191,192]. Moreover, the main opponent of biosensors are immuno-sensors, which are mostly and commonly utilized to determine various important substances and support excellent specificity and sensitivity analysis at a low price. Currently, biosensors which have been commercially available provide only few applications, mostly bio-chemical control, mainly targeted at scientific and research laboratories. Due to sensitivity, durability, stability, low cost and their ease of use, SPR sensors dealing with current technologies have a main challenge to develop into commercial sensing devices.

Presently, SPR biosensors are restricted to around 1.0 pg mm^−2^ for the detection of biomaterials which is insufficient to identify low molecular weight analyte concentrations [193,194,195,196,197,198,199]. Sophisticated data-processing methods can improve the SPR sensor resolution limit for a lower size of biomolecules, and at present there is no other method present to enhance the detection limit. SPR technology would be useful in the future, taking advantage of optical waveguide technology, which provides the design of compact minimized and rough-sensing elements with the perspective of assembling several sensors on one chip [200,201,202,203].

## 8. Commercial SPR Biosensors

Biacore has established a series of laboratory SPR instruments (Biacore 1000, Biacore 2000, Biacore 3000, Biacore C, Biacore J, Biacore Q, Biacore X) for the following decade [204,205,206,207,208,209,210,211]. The Biacore S51 offers higher sensitivity and higher throughput, which was developed most recently. Nippon Laser, British Windsor Scientific (IBIS) and Electronics Laboratory (Analytical μ-Systems (BIO-SUPLAR, SPR-670 and SPR-CELLIA, Texas Instruments (Spreeta), Dallas, TX, USA) have developed further SPR sensors [212,213,214,215,216,217,218,219,220].

## 9. Conclusions

The present paper comprehensively reports the latest technological advancement in SPR sensors based on 2D materials. This paper also highlights the fundamentals of surface plasmon, along with selected research work on SPR sensors based on 2D materials, published during the last ten years (2010–2020). It has been observed that the heterostructure of graphene, black phosphorus (BP) and TMDC-based SPR biosensors provide 2.5 times more sensitivity as compared to conventional biosensors. For biosensing and industrial applications, 2D materials have great potential to develop the most effective SPR sensors. Out of all the 2D materials, graphene has come to light as a paramount material for developing SPR-based sensors due to its magnificent electrical and optical properties. To improve sensor performance as well as the protection of metal layers from oxidation issues, the researcher uses a graphene layer with metal layer. The high surface to volume ratio is favourable for adsorption improvement on the sensors surface, which results in significant improvement in sensing performance. Thus, the 2D materials have great potential to develop high-performance SPR sensors for biosensing and industrial applications.

## Figures and Tables

**Table 1:** The sensitivity range comparison of surface plasmon resonance (SPR) and localized surface plasmon resonance (LSPR) [22].

Sensitivity	SPR	LSPR
Refractive index sensitivity (nm/RIU)	10^6^	10^2^
Distance dependence (nm)	1000	10
Temperature control	Yes	No
Simple instrumentation	No	Yes

**Table 2:** Basic electronic characteristics of the various transition metal dichalcogenides (TMDCs) measured both experimentally and theoretically.

Electronic Properties	MoS_2_	MoSe_2_	WS_2_	WSe_2_	Reference
Optical bandgap E_g_ (eV)	~2.0	~1.70	~2.10	~1.75	[36,37,38,39,40,41]
Exciton binding energy(eV)	~0.20–0.90	~0.50–0.60	~0.50–0.70	~0.40–0.45	[42,43,44,45,46,47,48]
Spin–orbit splitting in Conduction band (meV)	~−3.0	~−20.0	~−30.0	~−35.0	[49,50]
Spin–orbit splitting in Valance band (meV)	~150.0	~180.0	~430.0	~470.0	[51,52,53]
Band masses (m_o_)	~0.50	~0.60	~0.40	~0.40	[54,55,56,57,58,59,60]

**Table 3:** Some refractive index database of prisms at a wavelength of 633 nm taken from the database of SCHOTT (Mainz, Germany) optical glass [91].

Prism Type	Wavelength (nm)	Refractive Index (n_c_ = n + ik)
CaF2	633	1.4329
BK7	633	1.5151
BAF10	633	1.6671
BAK1	633	1.5704
SF5	633	1.6685
SF10	633	1.7231
SF11	633	1.7786
FK51A	633	1.4853
LASF9	633	1.8449

**Table 4:** Values of the refractive index and dielectric constants for the different metals.

Metals	Wavelength (nm)	Refractive Index (n_c_ = n + ik)	Dielectric Constant (ε_n_ + iε_k_)	Ratio (ε_n/_ε_k_)	Reference
Silver (Ag)	633	0.2184 + 3.5113i	−18.22 + 0.48i	38.00	[96]
Gold (Au)	633	0.1726 + 3.422i	−10.92 + 1.50i	7.34	[97]
Copper (Cu)	633	0.5840 + 3.6466i	−14.67 + 0.72i	20.40	[98]
Aluminium (Al)	650	-	−42.00 + 16.41i	2.55	[99]

**Table 5:** Values of the refractive index and dielectric constants for the different 2D materials.

2D Materials	Wavelength (nm)	Monolayer Thickness (nm)	Refractive Index (n_c_ = n + ik)	Dielectric Constant (ε_n_ + iε_k_)	Ratio (ε_n/_ε_k_)	Reference
Graphene	633	0.34	3.0 + 1.1487i	7.68 + 6.89i	1.114	[111]
Black Phosphorus (BP)	633	0.53	3.5 + 0.01i	-	-	[111]
MoS_2_	633	0.65	5.0805 + 1.1724i	24.4368 + 11.9122i	2.05	[112]
MoSe_2_	633	0.70	4.6226 + 1.0062i	20.3560 + 9.3040i	2.19	[112]
WS_2_	633	0.80	4.8937 + 0.3123i	23.8511 + 3.0580i	7.80	[113]
WSe_2_	633	0.70	4.5501 + 0.4332i	20.5156 + 3.9423i	5.20	[113]

**Table 6:** Comparison of the sensitivity of the differently reported SPR sensors based on 2D material.

Configuration	Wavelength (nm)	Sensitivity (°RIU^−1^)	References
Prism/Au/Si/Graphene	633	30.42	[119]
Prism/Au/Graphene/Affinity Layer	633	33.98	[120]
Prism/Au/Si/MoS_2_/Graphene/BRE	632.8	50.33	[46]
Prism/Au/Si	632	106.29	[121]
Prism/Au/Si/MoS_2_	633	131.70	[122]
Prism/Au/MoS_2_/Au/Graphene	633	182.00	[123]
Prism/Au/BP	633	180.00	[124]
Prism/Au/Si/MoS_2_/Au/Graphene	633	210.00	[125]
Prism/Graphene/WS_2_	633	95.71	[126]
Prism/Au/MoS_2_/Graphene hybrid	633	89.29	[127]
Prism/Au/MoS_2_/Ni/Graphene	633	229.00	[128]
Prism/Blue phosphorene/MoS_2_	632.8	150.66	[129]
Prism/TiO_2_/SiO_2_/Ag/MoS_2_/Graphene	633	98.00	[130]
Prism/Ag/Franckite/Graphene	633	196.00	[131]
Prism/Au/SnSe/Graphene	633	94.29	[132]

**Table 7:** Advantages and disadvantages of the different techniques.

Method	Advantages	Disadvantage
Physical vapor deposition (PVD)	Aesthetic and corrosion properties, wear and corrosion resistance, deposition of thin film possible and adjustable	Corrosion resistance is affected by abrasion, requires a high vacuum, for polymer deposition applications degradation control is challenging
Chemical vapor deposition (CVD)	Deposition of various CVD types of materials with different microstructures; corrosion and wear resistance, works with atmospheric and low pressures	Need for heat-resistant substrates, ultra-high vacuum, less material wastage
Sol-gel	High adhesion, ability to coat complex geometries, biomedical applications, gives ion release and corrosion protection, flexibility in the composition; cost effective, multi-layered coating possible, no need for conductive substrates	During the heat treatment, failure of coatings possible on multi-layered coating structures, slow rate of coating cycle, thickness control
Sputtering	Better crystallinity and control on deposition rate	Produce multiple phases, high operational cost
Electro deposition screen printing	Atmospheric temperature deposition, low-cost method	Process optimization is difficult
Spray coating	Low cost, high throughput, scalable	During spray coating precursor material wastage
Spin coating	Easy operation, film uniformity (lab scale), low cost,	More material wastage, no uniformity over a large area, roll to roll incompatible
Doctor’s blade	Roll to roll compatible, less material wastage, better stoichiometric control	Accumulation due to slow solvent evaporation
Molecular beam epitaxy (MBE)	Useful for phase segregation and defect studies, because of ultra-high vacuum deposition, minimum contamination	No report on large area deposition and high efficiency
MOCVD	Growth rate is faster than MBE, useful for basic studies	No report on large area growth, not suitable for industrial processes, process is not abrupt as MBE
Electron beam deposition	Film purity and good stoichiometry	No report on large area deposition, incompatible with industrial processes.
Pulsed laser deposition	Binary phase can be neglected, good stoichiometric, target composition can be transferred to films, binary phase can be avoided,	Not suitable for large area, no report on large area, stoichiometric
Inkjet printing	compatible with roll to roll technology, mask less patterning simplifies processing steps,	Low efficiency

**Table 8:** Advantages and disadvantages of various biosensors.

Type of Biosensors	Classification	Advantage	Disadvantage
Electrochemical biosensors	Impedimetric; Conductometric; Potentiometric; Amperometric;	Good resolution, excellent accuracy, repeatability	Susceptible to the temperature changing, short shelf life
Optical biosensors	Surface plasmon resonance (SPR)	High sensitivity, remote controllable	Costly, fragile
Acoustic wave biosensors	Mass based	Highly sensitive to minor mass changes, detection of molecules that do not have electrically conducting property nor optical signal (e.g., virus)	Fragile, mechanically unstable

**Table 9:** List of applications of the fibre-optic SPR biosensors.

Analyte	Technique	Biosensor	Lod/Sensitivity	References
SEB	SPR	Immunoassay	0.50 ng/mL	[146]
CEA	SPR	Immunoassay	0.50 ng/mL	[147]
Salmonella	SPR	DNA	0.50 nM	[148]
TNF-α	SPR	DNA	0.68 pM	[149]
Hunan IgE	SPR	DNA	2.0 nM	[150]
Urea	SPR	Enzyme	10^−4^–10^−1^ M	[151]
Pesticide	SPR	Enzyme	-	[152]
RBL-2H3	SPR	Cell	-	[153]
Peripheral-B	SPR	Cell	-	[154]
DNP-HSA	SPR	Cell	-	[153]
HEK-293	SPR	Cell	-	[155]
